# Genome Sequence of *Youngiibacter fragilis*, the Type Strain of the Genus *Youngiibacter*

**DOI:** 10.1128/genomeA.01183-13

**Published:** 2014-01-23

**Authors:** Colin B. Wawrik, Amy V. Callaghan, Blake W. Stamps, Boris Wawrik

**Affiliations:** a Franziskanergymnasium Kreuzburg, Großkrotzenburg, Germany; b Department of Microbiology and Plant Biology, University of Oklahoma, Norman, Oklahoma, USA

## Abstract

The genome of *Youngiibacter fragilis*, the type strain of the newly described genus *Youngiibacter*, was sequenced. The genome consists of 3.996 Mb, with a G+C content of 46.6 mol%. *Y. fragilis* originates from coal-bed methane-produced water and may provide insight into the microbiological basis of biogas production in coal beds.

## GENOME ANNOUNCEMENT

*Youngiibacter* is a newly described genus of *Clostridiaceae* (1). Its type strain, *Youngiibacter fragilis*, is a strictly anaerobic, Gram-negative, non-spore-forming rod that ferments a range of carbohydrates to ethanol, formate, acetate, and CO_2_. It was isolated from produced water of a coal-bed methane (CBM) production well in the Beluga River Gas Field, AK. CBM production emerged in the 1980s and has since become an important source of natural gas in the United States, China, Japan, Australia, and New Zealand (2). Geochemical and microbiological investigations have repeatedly demonstrated that biological production of methane plays an important role in the generation of methane in coal formations (2, 3). It is thought that a diverse microbial community mediates the initial activation of the complex coal matrix, and that subsequent fermentations of degradation products are coupled to the production of methane via close interactions between bacteria and methanogenic archaea (2). The biochemical mechanisms that govern anaerobic hydrocarbon activation and degradation are an area of active research. Genomic analysis of bacterial and archaeal isolates from such environments may shed light on the role that individual microbial community members play in subsurface organic matter transformations.

Whole-genome shotgun sequencing of *Y. fragilis* was conducted via Illumina MiSeq technology by generating a library with 350-bp inserts and sequencing 250-bp paired-ends. A total of 4.68 × 10^6^ paired-end reads were obtained, yielding 2.34 × 10^9^ bp of raw sequences. Reads were trimmed by removing adapters, retaining sequence data with quality scores of >30. The surviving paired-end reads with sequence lengths of >100 bp (97.8% of reads) were assembled using CLC Genomics Workbench (CLC bio, Cambridge, MA). Contigs longer than 500 bp were considered for further analysis. Scaffolding of contigs was attempted using SSPACE (4) but did not improve the assembly. Assembly produced 242 contigs at ca. 350× coverage. The *N*_50_ of the assembly is 41,656 bp, and the longest contig is 216,900 bp. The 16S rRNA gene was reconstructed from reads using EMIRGE (5), yielding a single 16S rRNA gene that was 100% identical to the known sequence of *Y. fragilis* (1). No evidence of plasmids was observed.

Annotation was conducted using the Rapid Annotation using Subsystem Technology (RAST) pipeline (http://rast.nmpdr.org). The genome contains 3,722 predicted proteins in 374 subsystems. Among genes with predicted functions, 23% are dedicated to carbohydrate metabolism, consistent with the phenotypic characterization of *Y. fragilis*. The genome lacks genes for dissimilatory sulfate reduction, nitrate reduction, flagellar motion, and chemotaxis. Given the origin of *Y. fragilis*, the genome was further investigated for the presence of genes involved in anaerobic hydrocarbon activation, with special focus on those relevant to coal degradation. Genes involved in known pathways for the anaerobic activation of alkanes, toluene, xylenes, ethylbenzene, phenol, acetophenone, benzene, and naphthalene were not detected. These data indicate that *Y. fragilis* is not likely to play a role in the initial activation of coal, but rather that it plays a role in the reprocessing and secondary fermentation of carbon in the subsurface.

### Nucleotide sequence accession numbers.

This whole-genome shotgun project has been deposited at DDBJ/EMBL/GenBank under the accession number AXUN00000000. The version described in this paper is version AXUN02000000.
